# *In-vitro* Activity of 10 Antifungal Agents against 320 Dermatophyte Strains Using Microdilution Method in Tehran 

**Published:** 2013

**Authors:** Parvaneh Adimi, Seyed Jamal Hashemi, Mahmood Mahmoudi, Hossein Mirhendi, Mohammad Reza Shidfar, Masood Emmami, Ali Rezaei-Matehkolaei, Mohsen Gramishoar, Parivash Kordbacheh

**Affiliations:** a *Department of Medical Parasitology and Mycology, Tehran University of Medical Sciences, Tehran, Iran.*; b *Department of Epidemiology and Biostatistics, School of Public Health, Tehran University of Medical Sciences, Tehran, Iran.*; c *Department of Medical Mycology, School of Medicine, Ahvaz Jundishapur University of Medical Sciences, Ahvaz, Iran. *

## Abstract

Dermatophyte fungi are the etiologic agents of skin infections commonly referred to as ringworm. These infections are not dangerous but as a chronic cutaneous infections they may be difficult to treat and can also cause physical discomfort for patients. They are considered important as a public health problem as well. No information is available regarding the efficacy of antifungal agents against dermatophytes in Tehran. Therefore, in this study we evaluated the efficacy of 10 systemic and topical antifungal medications using CLSI broth microdilution method (M38-A). The antifungal agents used included griseofulvin, terbinafine, itraconazole, ketoconazole, fluconazole, voriconazole, clotrimazole, ciclopirox olamine, amorolfine and naftifine.Fifteen different species of dermatophytes which were mostly clinical isolates were used as follows; *T. mentagrophytes, T. rubrum, E. floccosum, M. canis, T. verrucosum, T. tonsurans, M. gypseum, T. violaceum, M. ferruginum, M. fulvum, T. schoenleinii, M. racemosum, T. erinacei, T. eriotrephon and Arthroderma benhamiae*. The mean number of fungi particles (conidia) inoculated was 1.25 ×10⁴ CFU/mL. Results were read after 7 days of incubation at 28 °C. According to the obtained results,itraconazole and terbinafine showed the lowest and fluconazole had the greatest MIC values for the most fungi tested. Based on the results, it is necessary to do more research and design a reliable standard method for determination of antifungal susceptibility to choose proper antibiotics with fewer side effects and decrease antifungal resistance and risk of treatment failure.

## Introduction

Cutaneous mycoses are divided into two groups based on their etiologic agent, including dermatomycosis and dermatophytosis. Dermatomycosis are cutaneous mycoses caused by any fungi other than dermatophytes. Dermatophytes are a closely related group of fungi in 3 anamorphic genera of *Trichophyton, Microsporum* and *Epidermophyton* that originate from humans, different animals or soil. Except for a few systemic diseases and immunosuppressed patients (1-7), they usually invade only keratinized tissue, such as skin epidermis, nail plate and hair. Although there are approximately more than 40 different species in these three genera, most infections are due to *Trichophyton rubrum, Trichophyton mentagrophytes, Epidermophyton floccosum* and *Microsporum canis*. A single species may be seen in several disease types.

Griseofulvin was the only approved systemic antidermatophytic agent, however,at present new antifungal drugs with fewer side effects is available. Terbinafine (allylamines), ketoconazole (imidazole group), itraconazole, and fluconazole (triazoles group) are mostly used orally, and clotrimazole (imidazole), ciclopiro xolamine (substituted pyridine), amorolfine (morpholin) and naftifine (allylamines) are usually applied topically for the treatment of these infections. Treatment failure occurs in 25-40% of patients with onychomycosis and 6.5% of those with tineacorporis and tinea cruris (8). Although, role of drug resistance in treatment failure is not clearly known, *in-vitro *susceptibility testing could help the clinicians to select the proper antifungal agent.There is no standard method available for susceptibility testing of dermatophytes. Determination of MIC (Minimal Inhibitory Concentration) by CLSI dilution method (M38-A) has been suggested in several studies (3, 9-18). There are more than 33 articles published on epidemiology, clinical aspects and etiology of dermatophytosis from Iran. However, only few articles studing antifungal susceptibility with the different method for these fungi (19-24). According to CLSI protocol M38-A, completely synthetic medium RPMI1640 (without sodium bicarbonate and L-glutamine at pH 7.0) supplemented with 0.165M morpholinepropanesuphonic acid (MOPS) is suitable for broth dilution based antifungal susceptibility determination for filamentous fungi (15). However, there are differences in time and incubation periods, form and numbers of inoculated fungi and MIC breakpoint determination and culture media used for different experiments.

Correlations of MICs with clinical outcomes have not been determined (10). It seems that comparing relying on the crude results is difficult, thus biostatic methods offered calculation of some parameters, like geometric mean (GM). Definitely, the geometric mean is the nth root of products of the value in a series of *n*-values. The geometric mean is more useful and representative than the arithmetic mean when describing a series of reciprocal or fractional values. The geometric mean can be used only for positive values (25).

The purpose of this study was to determine the susceptibility of 10 oral and topical antifungal agents including fluconazole, itraconazole, ketoconazole, voriconazole, clotrimazole, terbinafine, naftifine, amorolfine, ciclopirox olamine and griseofulvin against clinical dermatophytes isolated from patients in Tehran, Iran. 

## Experimental

*Dermatophyte strain*

A total of 320 dermatophyte strains belonging to 15 species were recognized using conventional methods such as slide culture, growth in CMA with 1% glucose, sabouraud dextrose agar with 3% NaCl, hair perforation, growth at 37°C, urease activity, and Trichophyton agar. *T. eriotrephon*, and *Arthroderma benhamiae *were diagnosed based on sequencing.

Except *M. fulvum*, all other strains were clinical isolates from different laboratories in Tehran and included *T. rubrum *(n=89), *T. mentagrophytes *(n = 136), *E. floccosum *(n = 46), *T. verrucosum *(n = 11), *T. violaceum *(n = 2), *T.tonsurans *(n = 8), *M. canis *(n = 16),*M. gypseum *(n = 4), *M. ferruginum *(n = 2), *Arthroderma benhamiae *(n = 1), *M. racemosum *(n = 1), *T. erinacei *(n = 1), *M. fulvum *(n= 1), *T. schoenleinii *(n = 1) and *T. eriotrephon *(n = 1). Four standard strains cultures of *T. rubrum *(ATCC MRL 666), *Aspergillus fumigatus *(ATCC304305), *Candida krusei *(ATCC 6258), and *Candida parapsilosis *(ATCC 22019 ) were also used for quality control of tests. Prior to testing, the fungi were sub-cultured on sabouraud CAF agar with actidione (Liofilchem, Italy) at 28 °C for 7 to 15 days to ensure purity, and then on potato dextrose agar (PDA) (Liofilchem, Italy) for sporulation of the inoculum.Tests were performed using NCCLS(CLSI) broth micro dilution method (M38-A) (15), as coming below.

*Culture media*

All dermatophyte strains were on RPMI 1640 medium (Sigma – Aldrich Chemie, GmbH, Riedstr) with L-glutamine and without sodium bicarbonate buffered at pH 7.0 with 0.165 M morpholinepropanesulfonic acid (MOPS) (Sigma, Aldrich Chemie, GmbH, Riedstr).

*Antifungal agent dilution*

Crude powder of antifungal drugs, including fluconazole, ketoconazole, itraconazole, clotrimazole, ciclopirox olamine, terbinafine, griseofulvin (Sigma-Aldrich Chemical Company, GmbH, Riedstr), naftifin ehydrochloride (Toronto Research chemical Inc. Canada), amorolfine hydrochloride (Tokyo Chemical Industry Co. Toshima, KITA –KU, Tokyo, Japan) and voriconazole (Pfizer S.A Amboise Cedex, France), were preparedby dissolving the powders in their specific solvents. Fluconazole dissolved in distilled water, while other drugs dissolved in 100% dimethyl sulfoxide (DMSO, Sigma-Aldrich).

Final concentrations of antifungal drug s ranged between 0.0625 and 256 μg/mL for fluconazole, 0.0312 and 32 μg/mL for ketoconazole, clotrimazole,ciclopirox, and naftifine, 0.0078 and 16 μg/mL for voriconazole,0.0078 and 8 μg/mL for itraconazole, 0.0156 and 16 μg/mL for terbinafine, and 0.0312 and 256 μg/mL for griseofulvin.

*Inoculum preparation*

Stock inoculum suspensions of the fungi were prepared from 7 to 10 day old cultures grown on PDA at 28°C. Mature colonies were covered with approximately 5 mL of sterile saline (0.85%) by scraping the surface with the tip of a sterile swab. The resulting mixture of conidia and hyphal fragments was withdrawn and transferred to sterile tubes. Heavy particles were allowed to sediment for 15 to 20 min at room temperature.The upper suspension was transferred to another sterile tube and mixed with a vortex mixer for 15 sec. Before the turbidity of the supernatants was measured spectrophotometrically (Jenway Model 6305) at a wavelength of 530 nm, transmission was adjusted to 65 by ETO sterilized disposable cuvette. Each suspension was diluted 1:50 to 1: 100 in RPMI 1640 to obtain twice the final test inoculum concentration. The inocula corresponding to the strains were quantified by plating 5 μL of a 1:100 dilution of the adjusted inoculum on PDA plates and spread in 4 directions by a sterile loop. The plate contents were incubated at 30°C and observed daily for the presence of fungal colonies. Colonies were counted, when the growth became visible, as number of CFU per milliliter.

*Test procedure*

All the tests were performed in sterile, flat-bottomed, 96-well micro plates (Orange Scientific, E.U). Aliquots of 100 μL of the 2× drug dilutions were inoculated into the wells with a multichannel pipette. The micro plates were put in storage at  22°C until used.

For performing the susceptibility testing, 100 μL of the diluted inoculum suspensions was added to each well to bring the drug dilutions to the final test concentrations. Growth and sterility control wells were also prepared for each isolate tested. The micro plate contents were incubated at 28 °C, avoiding desiccation of the wells, and were read visually with the aid of an inverted reading mirror after 7 days of incubation. Every test was repeated at least twice.

*Reading results*

For fluconazole and griseofulvin, the MIC was defined as approximately 50% growth inhibition compared to the growth control well. For other antifungal agents, the MIC was defined as the lowest concentration showing 100% growth inhibition.

*Data analysis*

Geometric mean, MIC range, MIC50, MIC80 and MIC90 were obtained for all the isolates tested. MIC value of antifungal drugs for different species were compared by repeated measure and one-way ANOVA and post-hoc Scheffe tests using SPSS version 16 software. P-values < 0.05 were considered statistically significant.

## Results and Discussion

A total of 320 dermatophytes, mostly clinically isolated from patients, were tested using modified M38 –A CLSI micro dilution method*. T. mentagrophytes, *followed *by T. rubrum *and *E. floccosum, *were the most common dermatophytes isolated from patients in Tehran.

*In-vitro *activities of 10 antifungal agents that potentially can be used either orally or topically, following micro dilution and 7 day incubation at 28 °C are reported in Table 1. Geometric mean MICs, MIC range, MIC 50, MIC 80 and MIC 90 were obtained for all the isolates tested.

**Table 1:** *In-vitro *susceptibility of 10 antifungaldrugs against 15 species of dermatophytes

**Species(number ** **of strain tested)**		Concentration (μg/mL)									
		Terb	Gri	Itra	keto	Flu	Vor	Clz	Ciclo	Amor	Naft
*T. rubrum* (n = 89)	GM	0.172	1.61	0.06	0.67	11.05	0.19	0.34	1.52	0.316	0.182
	MIC50	0.0312	2	0.0625	1	32	0.125	0.25	2	0.25	0.125
	MIC80	8	128	0.5	4	128	1	1	8	2	0.5
	MIC90	16	256	2	8	256	4	4	16	4	4
	Range	0.0156-16	0.0312-256	0.0009-4	0.0312–32	0.0625–256	0.0078–8	0.0312-16	0.0312-32	0.0078–32	0.0625–16
*T. mentagrophytes* (n = 136)	GM	0.142	2.66	0.045	0.94	18.82	0.28	0.247	2.26	0.389	0.147
	MIC50	0.0312	2	0.0625	2	64	0.5	0.125	2	0.25	0.25
	MIC80	8	256	0.25	4	128	1	1	8	4	8
	MIC90	16	256	0.5	8	256	4	2	16	8	12
	Range	0.0156-16	0.0312-256	0.0009-4	0.0312-32	0.625-256	0.0156-8	0.0312-32	0.0312-32	0.0078-32	0.0625–16
*E. floccosum* (n = 46)	GM	0.093	2.31	0.035	0.43	10.44	0.174	0.33	1.81	0.245	0.326
	MIC50	0.0312	8	0.0312	0.5	32	0.125	0.25	2	0.125	0.125
	MIC80	1	128	0.5	4	256	1	2	8	2	8
	MIC90	5.6	256	0.5	8	256	2	4	16	4	22.4
	Range	0.0156-16	0.2-256	0.0009-4	0.0312-8	0.0625-256	0.0078-8	0.312-32	0.0312-32	0.0078-32	0.0625-32
*T. verrucosum* (n = 11)	GM	0.062	1.99	0.0186	0.77	16	0.108	0.266	6.62	0.87	0.31
	MIC50	0.0312	2	0.0076	1	64	0.125	0.5	8	1	0.0625
	MIC80	0.125	128	0.5	4	128	0.125	1	16	4	0.5
	MIC90	4	256	1	7.2	243	7.21	2	29	8	8
	Range	0.0312-8	0.0312-256	0.0009-1	0.0312-8	0.0625-256	0.0156-8	0.0312-2	0.0312-32	0.0625-8	0.0312-16
*T. tonsurans* (n = 8)	GM	0.088	24.24	0.147	0.79	13.92	0.39	0.297	3.17	0.39	2
	MIC50	0.0078	128	0.375	1	32	0.312	0.375	9	0.75	4.5
	MIC80	1.7	256	2	3.2	217.6	3.2	0.8	25.6	2	16
	MIC90	8	256	2	4	256	4	2	32	2	16
	Range	0.0156-8	0.03-256	0.0076-2	0.125-4	0.0625-256	0.0625-4	0.0625-2	0.0312-32	0.0078-2	0.125-16
*M. canis* (n = 16)	GM	0.044	0.16	0.08	0.31	11.98	0.164	0.20	1.64	0.183	1
	MIC50	0.0312	0.0312	0.0312	0.25	32	0.125	0.125	1	0.0625	0.5
	MIC80	0.0625	2	0.5	2	180	3.3	1	16	4	6.6
	MIC90	4	96	0.5	4	256	7.5	10.4	32	16	8
	Range	0.0312-8	0.02-128	0.0009-0.5	0.0625-4	0.625-256	0.0156-8	0.0312-16	0.0615-32	0.0078-16	0.5-8
*M. gypseum* (n = 4)	GM	0.044	2.82	0.051	3.36	45.25	0.353	0.176	1.68	0.297	0.125
	MIC50	0.046	128	0.25	3	40	0.5	0.125	1.5	0.125	0.125
	MIC80	0.125	256	2	16	256	1	1	16	8	0.25
	MIC90	0.125	256	2	16	256	1	1	16	8	0.25
	Range	0.0625-0.5	0.0312-256	0.0009-2	1-16	16-256	0.125-1	0.0625-1	0.25-16	0.0625-8	0.0625-0.25
*T. violaceum* (n = 2)	GM	0.044	16	0.25	0.25	11.31	0.031	0.25	0.70	0.0312	0.125
	Range	0.0156-0.125	16	0.25	0.25	1- 128	0.0312	0.125- 0.5	0.0312- 32	0.0312	0.0625-0.25
*M. ferruginum* (n = 2)	GM	0.35	2.82	0.043	0.5	2.82	1.41	1.41	2	1	1
	Range	0.25-8	0.0312-256	0.0076-0.25	0.0312 - 8	0.0625-128	0.5-4	0.5-4	1-4	0.5-2	0.125 - 8
*M. racemosum* (n = 1)	MIC	1	0.0312	0.0156	4	0.0625	0.25	16	1	0.0078	-
*M. fulvum* (n = 1)	MIC	0.0625	0.5	0.5	2	0.0625	0.0312	0.0625	0.0625	0.25	0.125
*T.schoenleini* ( n = 1 )		MIC	0.0156	1	0.0039	0.0312	0.0625	0.0078	0.0625	0.0625	0.0312	0.0156
*T.erinacei* ( n=1 )		MIC	0.0156	0.125	0.0625	0.0312	4	0.0625	0.5	0.0312	0.0625	0. 125
*T. eriotrephon* (n=1)		MIC	0.0625	0.0312	0.0039	2	128	0.125	0.25	0.0312	0.25	-
*Arthroderma benhamiae* ( n = 1 )		MIC	0.0156	1	0. 25	16	32	1	0.0625	16	0.25	-
*Total*		GM	0.131	1.96	0.047	0.74	13.62	0.22	0.28	2.05	0.33	0.38
		MIC50	0.0312	2	0.0625	1	48	0.125	0.25	2	0.25	0.125
		MIC80	2	128	0.5	4	128	1	1	8	2	4
		MIC90	8	256	0.5	8	256	4	2	16	4	8
		Range	0.0156-16	0.02-256	0.0009-4	0.0312- 32	0.0625-256	0.0078-8	0.0312-32	0.0312-32	0.0078-32	0.0625-32

Abbreviations: terb,terbinafine;Gri,griseofulvin;Itra,itraconazole;Keto,ketoconazole;flu,fluconazole;Vor,voriconazole;Clz,clotrimazole;Ciclo,ciclopirox olamine;Amor,amorolfine;Naft,naftifine;GM,geometric mean;MIC50,MIC80,MIC90,MIC at which 50,80 and 90% of fungi are inhibited respectively.

Geometric mean (GM) of itraconazole and terbinafine was lowest for *T. rubrum, T. mentagrophytes *and *E. floccosum *indicating that these drugs are most potent in *in-vitro *studies. Mean MICs of antifungal drugs did not show statistically significant differences between different species (p > 0.05).Voriconazole was more effective than ketoconazole, however fluconazole and griseofulvin had the lowest MIC against these 3 dermatophytes. Amongst topical antifungal agent, clotrimazole showed lowest MIC value followed by amorolfine, naftifine and ciclopirox olamine. 

Itraconazole and terbinafine showed the lowest GM MIC for *M. canis*.Griseofulvin had GM MIC lower than voriconazole, followed by ketoconazole and fluconazole against this microorganism. After fluconazole, griseofulvin had the highest GM MIC for mostother dermatophytes.Between 4 topicalantifugal agents, clotrimazole had the lowest MIC and GM MIC. Amorolfinewas the second antifungal agent against *M. canis*, and naftifine and ciclopirox olamine were rated third and fourth respectively.

Although terbinafine had the greatest *in-vitro *activity and lowest GM MIC against *T. tonsurans, M. gypseum, Arthroderma benhamiae*, voriconazole had the lowest MIC against *T. violaceum*. A surprising result was that itraconazole was the most potent antifungal agent against most of the microorganisms tested in this study.

These tests were performed under variable conditions in terms of type of culture media for sporulation, incubation temperature, number and type of inoculated fungi and method, reading and interpreting the result by different researchers.Thus it would be difficult to compare the results.

The culture media used for sporulation in this study was the PDA media which was in according with many other studies like Li *et al*., Santos *et al*., Sarifakioglu *et al*., Galuppi *et al*., Ghannoum *et al*, Carrillo *et al*, Fernandez Torres *et al*, and Esteban *et al. *studies (8, 10, 12, 14, 16, 26-29).

A 7-10-day incubation time was allocated for sporulation of the majority of dermatophytes, like *T. mentagrophytes *and *T. rubrum*. Longer periods up to 6 weeks were required for other fungi, especially *T. schoenleinii *and *T. violaceum *until the colony morphology changed to a white fluffy appearance. The incubation time used in this study was similar to the one used by Barro set *et al*. (30), Santos *et al*. (27), and Fernandez Torrez *et al*. (12, 14). 

For inoculation of fungal suspension into the 96 well microplates containing antibiotics, the suspension was diluted 1:50 with 65% transmittance at 530 λ wavelength which was in accordance with the instructions given by Esteban *et al*., and Fernandez Torres *et al. *(12, 29).

The results of micro dilution tests for most strains read after 7 days at 28 °C, when adequate growth was observed in the control well with significant opacity. The 7 day time period has also been mentioned in Santos *et al. *(27), Gupta *et al. *(31), Fernandez-Torres (14), and Barros (30) studies. This time period was shorter (4 days at 35°C) in Ghannoum *et al. *(10), and Mukherjee (32)) studies. This difference might be explained by the different temperatures used. Galuppi *et al*.(28) reported a longer period of 14 days and incubation at 30°C. This difference in the required incubation time may be due to the different volumes of fungi inoculated into the micro plates.

Antibiogram results for 320 dermatophytes showed that the highest susceptibility of dermatophytes was to itraconazole and the lowest to fluconazole. Our findings about susceptibility of dermatophytes to fluconazole is compatible withSantos *et al*. (27), Favre *et al. *(11), Barros *et al*. (30) and Sarifakioglu *et al.*(8) studies.Korting *et al*.(33) suggestedthat high values of MIC for fluconazole may be due to technical problems,such as interference with someingredients of the culture media or insolubility at high concentrations. 

However, geometric means MIC (GM) obtained in this study showed that the results of terbinafine for all species of dermatophytes were significantly greater than results of Gupta *et al, *study (31), but were in agreement with Munoz *et al*., Esteban *et al*. and Fernandez-Torres *et al*. (12, 16, 29) findings. The GMs of dermatophytes in Favre *et al*., and Galuppi *et al*. study were greater than our study (11, 28). In this study, the GMs of *M. gypseum, M. canis *and *T. violaceum *were similar (GM=0.04) and they were considered the most sensitive dermatophytes, whereas *T. rubrum *(GM=0.172) and *T. mentagrophytes *(GM=0.142) had the lowest susceptibility. In Esteban *et al*. (29) study, *T. rubrum *(GM=0.190) had the lowest susceptibility, while in Fernandez-Torres *et al. *study, *T. rubrum *and *T. violaceum *(GM=0.01) were the most sensitive dermatophytes (12). Considering the fact that laboratory methods were similar in these studies and also for determining the MIC endpoint for terbinafine is usually selected a well with 100% growth inhibition, it appears that the reported differences might be due to the differences between strains or different number of fungi tested.

In the present study, *T. tonsurans *(GM=24.24) showed the lowest and *M. canis *(GM=0.16) showed the highest susceptibility to griseofulv in. This is in complete agreement with Seebacher *et al, *opinion, and they suggest that griseofulvinis the best choice in the treatment Tinea capitis cases due to *M. canis *(18). *T. mentagrophytes *and *E. floccosum *have much lower susceptibility to griseofulvin than *T. rubrum*. Chadeganipour *et al. *(24) also reported higher MIC value for *T. mentagrophyes *than other tested dermatophytes. The GM reported by Galuppi *et al. *was also higher than our finding (GM=2.20) (28). However, Favre *et al. *reported an even smaller value (GM=0.37)(11). These differences are justifiable considering different readings/interpretations as 50%, 100% and 75% growth inhibition, respectively, compared to the growth control well of micro plate for determining MIC.

Itraconazole MIC results showed that GMMIC of *T. rubrum *in our study (GM=0.06) was much smaller than Gupta *et al*. (GM=0.143), Fernandez-Torres *et al*. (GM=0.09), Esteban *et al*. (GM=0.912), and Galuppi *et al. *(GM=0.96) findings. Gupta *et al*, and after that Fernandez - Torres *et al*. (31) were obtained the lowest GM for *T. mentagrophytes*. We also found the lowest GM for *T. mentagrophytes*. Esteban *et al*., followed by Galuppi *et al*, found the highest GM for this fungi (12, 28, 29, 31). 

Susceptibility of *T. rubrum *with GM=0.67 to ketoconazole in this study was almost similar to Fernandez -Torres *et al*, (GM=0.65) and Galuppi *et al*, (GM=0.76) study, but was significantly higher than those obtained by Favre *et al*, (0.22), Fernandez *et al*. (0.14) and Gupta *et al*. (0.165)(11, 12, 14, 28, 31, 34).

In the present study, *T. mentagrophytes *after *M. gypseum *showed the lowest and *E. floccosum *had the highest *in-vitro *sensitivity to fluconazole.

The GM in our study was much greater than the GMs obtained in Favre *et al. *(11), (GM=6.3) and Carrillo *et al*. (16), (M=15.08) study. In Carrillo *et al*. (16) study, *M. gypseum *and *T. mentagrophytes *had the highest GM which means the lowest susceptibility. Kotring *et al*. (33) showed that the concentration of this drug, following consumption of 150 mg orally after 7 days, reaches to 7.1 μg/mL in stratum corneum. In addition,MIC > 64 μg/mL is attributed to resistance to this drug (35, 36). MIC mean values for half of the dermatophyes strains in our study is about 7 times greater than *in-vivo *condition, used for treatment of dermatophyte infections. In Foroumadi *et al. *(37) study, high MIC value were seen in cases of vulvovaginal candidiasis in Tehran treated with fluconazole, too.

In this study, we examined voriconazole antifugal susceptibility against dermatophytes. However, this drug is not currently being used due to its high cost. According to our study*, T. schoenlinii, T. violaceum, T.verrucosum, M. canis, E. floccosum, T. rubrum, T. mentagrophytes, M. gypseum *and *T. tonsurans *had, in an ascending order, the lowest to highest susceptibility. The GMs for all strains were greater than those obtained in Carrillo *et al*, Favre *et al*, and Fernandez - Torres *et al*, studies. However, Fernandez-Torres *et al, *and Carrillo *et al*, showed the highest susceptibility of *E. floccosum *to voriconazole (11, 12, 14, 16, 38).

Among topical antifungal agents that are usually used along with systemic antifungal medications, we selected ciclopirox olamine, clotrimazole, amorolfine and naftifine for *in- vitro *antifungal susceptibility. Amorolfine and naftifine are not available in Iran and therefore are not prescribed. In our study, *M. gypseum *(GM=0.176) and *T. rubrum *(GM=0.34) had the highest and lowest susceptibility to clotrimazole, respectively. Fernandez-Torres *et al, *(14) found similar results and showed that *M. gypseum *was the most sensitive dermatophyte to clotrimazole. Whereas, in Fernandez - Torres *et al*, (12) study, *M. canis *along with *T. rubrum *had the highest susceptibility. Esteban *et al*. also reported different findings and indicated that *T. tonsurans *and *M. canis*had the highest susceptibility (29). This difference may be attributed to unequal size of inoculums.

Antibiogram of ciclopirox olamine showed that *T. violaceum *had the highest susceptibility, followed by *T.rubrum*, *M. canis*, *M. gypseum, E. floccosum, T. mentagrophytes*, *T. tonsurans*,*M.ferruginum *and *T. verrucosum *in a decreasing fashion.

In terms of quantity, MIC50 values in our study, except for *T. tonsura*ns, were in agreement with Korting *et al*. (33) findings. The figure obtained in our study was in the range of 0.125-2 μg/mL, while this was 2 or 3 μg/mL in Korting *et al, *study and both these rates were greater than figures obtained by Gupta *et al*, Santos *et al*, and Favre *et al*. studies (11, 27, 31). 

Susceptibility testing of dermatophytes to amorolfine has been previously performed by Li *et al *and Favre *et al*. (11, 26). The lowest values (MIC50=0.004, and MIC90=0.008 μg/mL) were reported by Favre *et al*, and these values were almost similar for 20 dermatophytes. Li *et al, *reported MIC50 and MIC90=0.04 μg/mL for *T. rubrum*, MIC50=0.04 and MIC90=0.8 μg/mL for *T. mentagrophytes *and MIC50=0.02 and MIC90=0.04 μg/mL for *E. floccosum*. Our findings for MIC50 and MIC90 for all strains were greater than the mentioned rates. The lowest rate of MIC50 belonged to *M. canis *(MIC50=0.0625 μg/mL) and the highest was reported for *T. verrucosum *(MIC50=1 μg/mL). The lowest MIC90 belonged to *T. tonsurans *(MIC90=2 μg/mL) and the highest to *M*. *canis *(MIC90=16 μg/mL). Thus, *M. canis *showed the widest MIC range.

Naftifine is a topical antifungal agent chemically belonging to the family of allylamines. For naftifine, the lowest value of MIC50 (MIC50=0.0625 μg/mL) belonged to *T. violaceum *and the highest to *T.tonsurans *(MIC50=4.5 μg/mL). MIC50 values for *M. gypseum, E. floccosum, T. mentagrophytes *and *T*. *rubrum *were exactly the same, but the MIC90 values were different from each other and the highest MIC90 belonged to *E. floccosum *(MIC90=22.4 μg/mL), followed by *T. tonsurans *(MIC90=16 μg/mL). 

In conclusion, the present study reveals that 2 out of 10 antifungal agents used in this study had high MIC value against dermatophytes. The clinical significance of testing this group of fungi remains uncertain,and breakpoints with proven relevance have yet to be identified and approved by regulatory agency. Based on our results, griseofulvin and fluconazole for dermatophyte infection should be used with a greater caution.Controversial findings in our study and others point the necessity of establishing a standard method for antibiogram of dermatophytes to facilitate the selection of drug similar to what is routinely performed for yeasts and bacteria.
